# Choose Home: Effectiveness and Acceptability of a Novel Geriatric Home-based Program

**DOI:** 10.1093/geroni/igaf122.4327

**Published:** 2025-12-31

**Authors:** Caroline Madrigal, Rebecca Howe, James Rudolph, Meghan Makela, Alexandra Nothern, Wendy Abrams, Jane Driver

**Affiliations:** VA Boston Healthcare System, Boston, Massachusetts, United States; VA Providence Healthcare System, Providence, Rhode Island, United States; VA Providence, Providence, Rhode Island, United States; VA Boston Healthcare System, West Roxbury, Massachusetts, United States; VA Boston Healthcare System, Boston, Massachusetts, United States; VA Boston Healthcare System, Boston, Massachusetts, United States; VA Boston Healthcare System, Brockton, Massachusetts, United States

## Abstract

Older Veterans prefer to ‘age in place’ in their homes/communities. However, complex, chronic medical conditions and limited resources put Veterans at risk for avoidable acute care use and long-term institutionalization. VA Boston Healthcare System developed Choose Home, an interdisciplinary, home-based program that provides intensive, short-term support via telephone and in-home medical and psychosocial case management. The program is designed to stabilize high-risk Veterans in their homes, promoting independence and connecting them to needed services. To evaluate the program’s impact from Veterans’ and their caregivers’ perspectives, we collected anonymous surveys at program discharge (n = 68) and conducted in-depth, semi-structured interviews with 12 Veterans (including 8 caregivers). 84% (n = 57) of Veterans/caregivers felt they were doing better at home after program participation, 90% (n = 61) felt better able to manage their care independently, and 88% (n = 60) felt satisfied with the program. We used thematic analysis to identify key programmatic outcomes from Veterans’ viewpoints, including: 1) independence/aging in place, via providing supports that allow Veterans to maintain autonomy and dignity; (2) holistic care during health crises, by offering coordinated support to help Veterans and families navigate overwhelming medical events; (3) caregiver empowerment, through education, respite, and practical assistance to mitigate burnout. Veterans’ perceptions of how Choose Home impacted their trajectory provide important insights into the program’s mechanisms of success which are essential for sustainment and scalability. Findings reflect the perceived effectiveness and acceptability of the program and highlight the value of integrated, community-based care models in enhancing the well-being and autonomy of Veterans and their caregivers.

